# A comparison of the sensitivities of detection of *Plasmodium falciparum *gametocytes by magnetic fractionation, thick blood film microscopy, and RT-PCR

**DOI:** 10.1186/1475-2875-8-98

**Published:** 2009-05-11

**Authors:** Stephan Karl, Timothy ME Davis, Tim G St-Pierre

**Affiliations:** 1 School of Physics, M013, The University of Western Australia, 35 Stirling Highway, Crawley WA 6009, Australia; 2 School of Medicine and Pharmacology, The University of Western Australia, Fremantle Hospital, Alma Street, Fremantle, WA, Australia

## Abstract

**Background:**

The magnetic properties of *Plasmodium*-infected erythrocytes have been exploited for different clinical and research purposes. A recent study in a rural clinical setting in Papua New Guinea has demonstrated that *Plasmodium falciparum *gametocyte detection is facilitated by magnetic deposition microscopy but no study has yet determined the relative sensitivity and limit of detection of a magnetic fractionation technique. The present study compares the detection limit and sensitivity of a technique based on the use of commercially available magnetic fractionation columns with those for thick blood film microscopy and reverse transcriptase polymerase chain reaction (RT-PCR) methods.

**Methods:**

Gametocyte detection in six series of dilutions of cultured *P. falciparum *parasites with known gametocytaemia was conducted using magnetic fractionation, thick blood film, and RT-PCR techniques.

**Results:**

The preparations obtained by the magnetic fractionation method were of thin film quality allowing easy gametocyte identification by light microscopy. Magnetic fractionation had a higher sensitivity and approximately two orders of magnitude better limit of detection than thick blood film microscopy. Gametocytes were also more readily detectable on the magnetically fractionated preparations. Magnetic fractionation had a similar limit of detection to that of RT-PCR.

**Conclusion:**

Magnetic fractionation is a highly sensitive and convenient method for gametocyte detection in comparison with the standard thick blood film and RT-PCR methods, and could readily be adapted to field application.

## Background

Based on the estimated average blood meal of *Anopheles gambiae *of 1.6 μL, and the observation that male gametocytes are less numerous than female gametocytes, the lower limit of male gametocyte density in human blood that has epidemiological implications for transmission is estimated to be around 2.5 μL^-1 ^[1]. It has also been reported that gametocyte densities of 1–10 μL^-1 ^can result in mosquito infection [2]. Detection and gender differentiation of gametocytes on standard thick blood films (TBF) at these densities is a challenge even for the experienced microscopist. For confident detection of low-level gametocytaemia molecular methods based on PCR need to be used [3].

Magnetic fractionation (MF) techniques can be used to enrich the concentration of erythrocytic stages of malaria parasites that contain haemozoin, the paramagnetic product of the haem detoxification pathway [4-6]. Commercially available MF columns have been used for various purposes in malaria studies ranging from simple reports confirming the feasibility of magnetically fractionating infected cells, to practical applications such as synchronization of cultures and sample preparation for immunological studies [7-9]. It has been demonstrated that the four main species of *Plasmodium *that infect humans can be concentrated by magnetic deposition microscopy (MDM), which utilizes a custom-made MF system that is being developed as a re-usable low cost alternative to the single-use MF columns [10]. It was shown recently that *Plasmodium falciparum *gametocyte detection can also be facilitated by MDM in a rural clinical setting in Papua New Guinea [11].

In the present study, the sensitivity and limit of detection of gametocytes by MF employing commercially available MF columns was compared with those of TBF and reverse transcriptase polymerase chain reaction (RT-PCR) methods. Each method was tested on gametocyte-containing parasite cultures serially diluted with whole blood. The preparations obtained with MF columns in this study are of thin film quality and allow easy identification and differentiation of gametocytes.

## Methods

The laboratory-adapted gametocyte producing *P. falciparum *strains 3D7, Dd2 and W2mef were cultured in RPMI 1640 HEPES (Sigma Aldrich) supplemented with 5 g/L albumax II (Invitrogen), 92.6 mg/L L-glutamine (Sigma Aldrich), 500 μg/L gentamicin (Sigma Aldrich), 50 mg/L hypoxanthine (Sigma Aldrich). Culture medium was changed once daily. Cultures were maintained at 4–5% haematocrit and diluted with red blood cells when parasitaemias exceeded 5%. Cultures were incubated in an airtight cabinet (Nalgene, Model 53170120) at 37°C in an atmosphere with approximately 5% oxygen concentration. The low oxygen atmosphere was generated by gassing the cabinet with a gas mixture (1% O_2_; 95% N_2_; 4% CO_2_) at 10–15 kPa for 60 seconds each time it had been opened. To induce gametocyte development, the parasite culture was allowed to grow to maximum parasitaemia with gametocytes appearing approximately 10 days later.

For the present experiments, 10-fold dilution series of the parasite culture were prepared in freshly drawn whole blood. The starting gametocytaemia was determined by counting the number of red blood cells per 100 gametocytes on a thin film prepared from the initial parasite culture. Six experiments were conducted comparing the sensitivities of MF, and RT-PCR (4 experiments using 3D7, 1 experiment using W2mef and 1 experiment using Dd2 strain). The total blood volume prepared for each dilution was 1610 μL of which 100 μL were used for MF, 10 μL to prepare TBF, and 1500 μL for RNA extraction and RT-PCR.

### Magnetic fractionations

Magnetic fractionations were conducted using a MidiMACS magnet, a MACS-multistand and LS columns (Miltenyi Biotech). The columns were placed into the magnet unit and primed with 0.7 mL sterile filtered magnetic fractionation buffer (MFB, PBS pH 7.4, containing 0.5 g/L bovine serum albumin and 0.0037 g/L EDTA) equilibrated to room temperature. 100 μL of blood from each dilution were suspended in 5 mL MFB in 15-mL centrifuge tubes (BD Biosciences) and incubated at room temperature for at least 10 min before the start of the fractionation. The flow rate through the column was regulated to 0.25 mL/min by attachment of a sterile syringe needle (BD Biosciences) with an inner diameter of 0.42 mm (26G) to the end of the column. After the cell suspension had passed through, the columns were washed with 2 × 1 mL MFB and removed from the magnet. The sterile needle was disconnected and the cells captured in the column were eluted into 15-mL centrifuge tubes (BD Biosciences) by washing with 1 × 5 mL MFB. These fractions were centrifuged at 400 *g *for 10 min (Sigma Laboratory Centrifuge, Model 4K15). The resulting cell pellet was resuspended in approximately 3 μL of PBS (pH 7.4) and spread on a microscope slide to form a circle of 0.5–1 cm in diameter. The slides were dried in an incubator (Sanyo, Model: MCO -15A) at 37°C for at least 30 min, fixed with methanol and stained with 5% Giemsa solution (Sigma-Aldrich) in PBS (pH 7.4) for 10 min. Leukocyte density was determined by counting the number of leukocytes in 100 high power fields across the MF preparation. Gametocyte density was determined by counting the number of gametocytes per leukocyte in 100 high power fields and assuming 8000 leukocytes per microliter of blood. If no gametocytes were detected in 100 fields, additional fields were examined until a gametocyte was seen or a maximum observation time of 60 min was reached.

### Preparation of thick blood films

Thick blood films were prepared from 10 μL of blood from each dilution. The films were stained with 5% Giemsa stain without prior methanol fixation for 10 min and carefully rinsed with water. The films were evaluated using the same protocol as that used for the MF preparations.

### Reverse transcriptase polymerase chain reaction

RT-PCR was conducted based on the methods of Mlambo *et al *[12], Babiker *et al *[13], and Menegon *et al *[14] for the gametocyte specific genes *Pfs25 *and *Pfg377 *using the same primer sequences as described in these publications. Total RNA was extracted from 1.5 mL blood from each dilution using the Qiagen Blood Kit following manufacturers instructions. RNA was eluted in 60 μL of RNAse free water. RNA was subjected to DNAse treatment (DNA-free Kit, Ambion Applied Biosystems). Total RNA was eluted with 60 μL of RNAse free water and DNAse treated using the Ambion DNA-free Kit (Ambion Applied Biosciences). The total RNA was quantified on a spectrophotometer (Nanodrop 1000, Thermo Fisher Scientific Inc.). RNA was subjected to reverse transcription directly without intermediate storage. cDNA was obtained from 5.5 μL of the total RNA eluate using reagents from a Superscript III first strand kit (Invitrogen) following manufacturers instructions with sequence specific primers *Pfs25*-R (5'-AATTCTTACATTATAAAAAAGCATACTC-3') for the *Pfs25 *gene and *Pfg377*-R3D1 (5'-GATGAAAGGGATATATCACCTCACAATGTG-3') and *Pfg377*-R3R2 (5'-GTCATGATTTTCTTCTCCTTCGGATATGG-3') for the *Pfg377 *gene respectively.

PCR was conducted immediately following reverse transcription using reagents from the Platinum Taq Polymerase Kit (Invitrogen). The first PCR was conducted using 2 μL of cDNA template in 18 μL of master mix containing primers *Pfs25*-R and *Pfs25*-F (5'-ATCGATATGAATAAACTTTACAGTTTGTTTCT-3') for *Pfs25 *and *Pfg377*-R3D1 and *Pfg377*-R3R2 for *Pfg377 *respectively. The nested PCR was conducted using 2 μL of the product from the first PCR. For *Pfs25 *primers *Pfs25*-1 (5'-TAATGCGAAAGTTACCGTGG-3') and *Pfs25-2 *(5'-TCCATCAACAGCTTTACAGG-3') were used. For *Pfg377 *primers *Pfg377-*R3D2 (5'-CCATAGGAATATTACACCATATCATGTG-3') and *Pfg377*-R3R1 (5'-TATGGTGATAAATGAGGAGTGTCCCCTTAC-3') were used. The first PCR was conducted on two separate PTC 100 Thermocyclers (MJ Research Inc.) using the cycling conditions for each gene as described previously by Babicker and Menegon with 35 cycles each [13,14]. No gels were run for the product from the first PCR and products were directly subjected to nested PCR. The nested PCR was conducted on a Rotorgene RG3000 thermocycler (Corbett Research) using the following cycling conditions for *Pfs25*: Hold: 95°C, 5 min; Cycling: 95°C, 15s; 50°C, 20s; 72°C, 40s; for 40 cycles; Hold: 72°C, 30s; Melt: 72°C to 99°C; Hold: 40°C, 30s and for *Pfg377*: Hold: 94°C, 5 min; Cycling: 94°C 35s, 55°C, 30s; 72°C, 60s for 40 cycles; Hold 72°C, 30s Melt: 72°C to 99°C; Hold: 40°C, 30s. PCR products were verified by melt curve analysis and gel electrophoresis in 1.5% agarose gel, containing ethidium bromide at 100 V for 60 min. The gels were visualized in a UV transluminator (Versadoc, Model 3000, Biorad).

### Data analysis

Only dilutions with a calculated gametocyte density of < 100 μL^-1 ^were used for comparison of the three techniques so that each of the six dilution series had one dilution in each of the following ranges: 10^1^–10^2^, 10^0^–10^1^, 10^-1^–10^0^, and 10^-2^-10^-1 ^μL^-1^. The limit of detection for each method in each dilution series was defined as the lowest gametocyte density where detection was positive. Detection limits were compared by using a nonparametric significance test (Wilcoxon's matched pairs test). The sensitivity of each technique for a given specified range of gametocyte density was defined as the fraction of gametocyte tests carried out in that range that were positive. In the case of RT-PCR a positive test was defined as the positive detection of at least one of the two genes being assayed.

## Results

**Figure 1:**
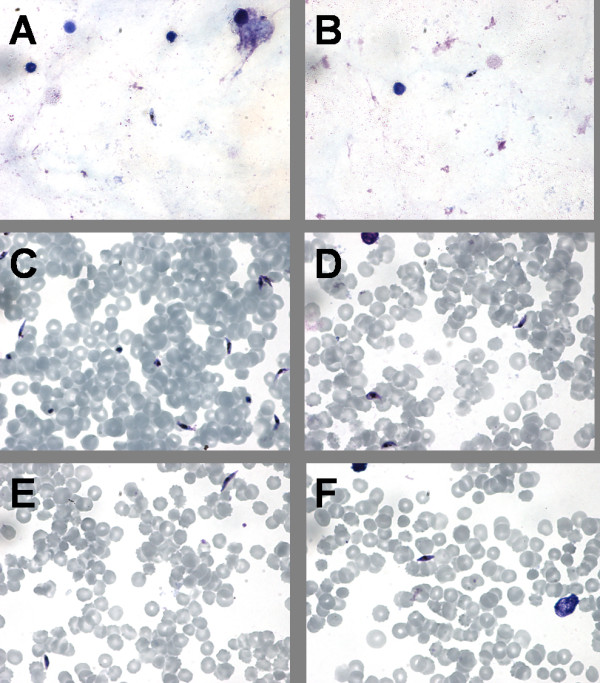
**Gametocytes on thick blood film (TBF) and magnetically fractionated (MF) preparations**. Gametocytes as observed on TBF in the ranges of 10^1^–10^2 ^(A) and 10^0^–10^1 ^(B) as compared with gametocytes as observed on MF preparations in the ranges of 10^1^–10^2 ^(C), 10^0^–10^1 ^(D), 10^-1^–10^0 ^(E) and 10^-2^-10^-1 ^(F). Images were obtained on a Nikon Eclipse TE2000 -N Microscope with a 1000 × optical magnification with a Nikon LH-M100CB-1 Camera.

Gametocytes were observed on the MF and TBF preparations as shown in Figure 1.

**Figure 2:**
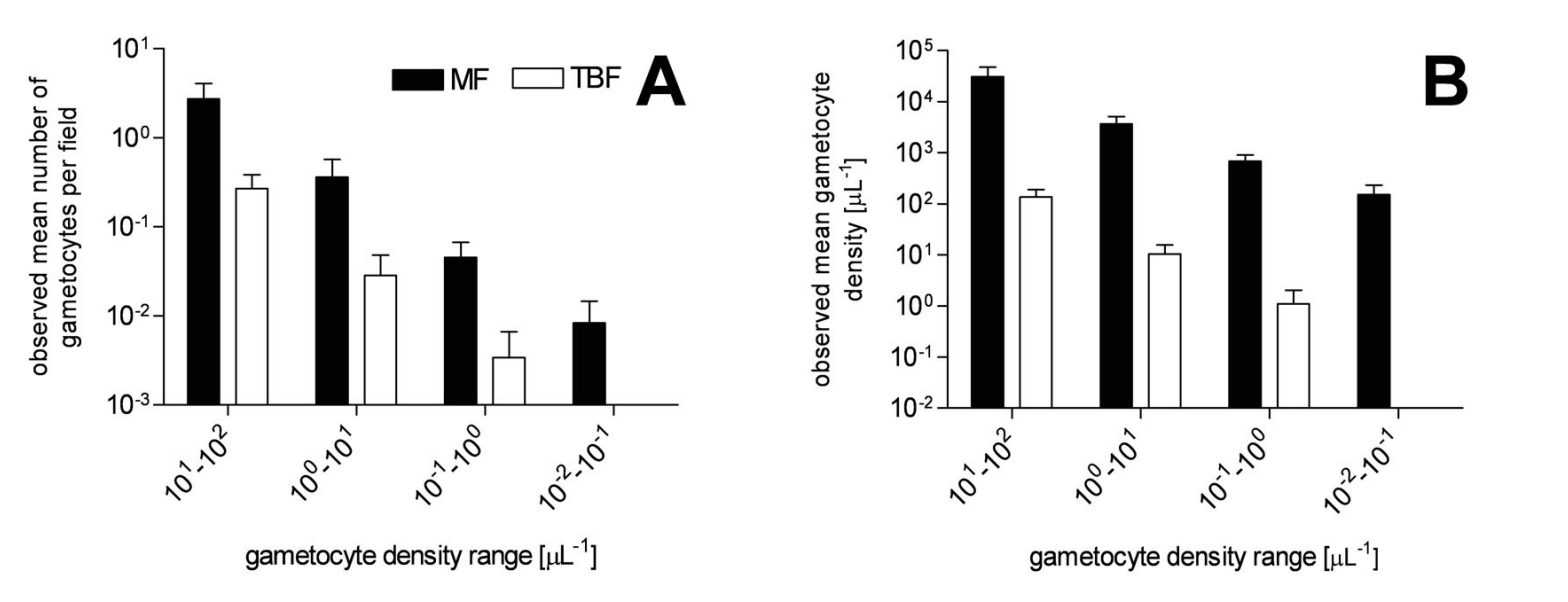
**Observed gametocyte increase on MF preparations as compared to TBF preparations**. Panel A: Mean gametocyte number per high power microscope field for each of the gametocyte density ranges for MF and TBF preparations. Panel B: Mean gametocyte density observed with MF and TBF for each of the gametocyte density ranges.

The increase in observed mean gametocyte number per field on the MF preparations was 12 (range 5–26) fold as compared to the corresponding TBF preparations, while the increase in observed mean gametocyte density was 319 (range 71–1387) fold (Figure 2).

The mean number of fields observed in 60 minutes for the gametocyte negative slides was 1740 (+/- 228) corresponding to a mean blood volume of 2.8 (SD 0.8) μL if based on 8000 leukocytes μL^-1^.

The mean total RNA from all 24 extractions was 79.9 (SD 20.1) ng/μL in 60 μL of RNAse free water. Parasite RNA did not make a difference to the mean RNA content, since leukocyte RNA formed the vast majority of the RNA present.

**Figure 3:**
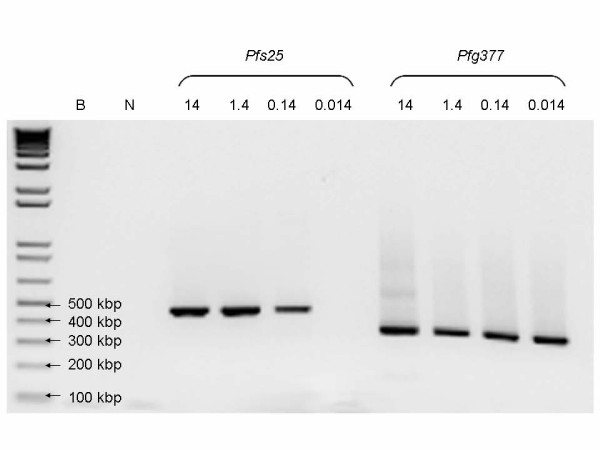
**RT-PCR for *Pfs25 *and *Pfg377***. Example gel showing bands for *Pfs25 *and *Pfg377 *from one of the dilution series used in this study. B = Blank, N = uninfected blood. The numbers above the other lanes are the gametocyte densities (μL^-1^) in the dilutions used in this experiment.

**Figure 4:**
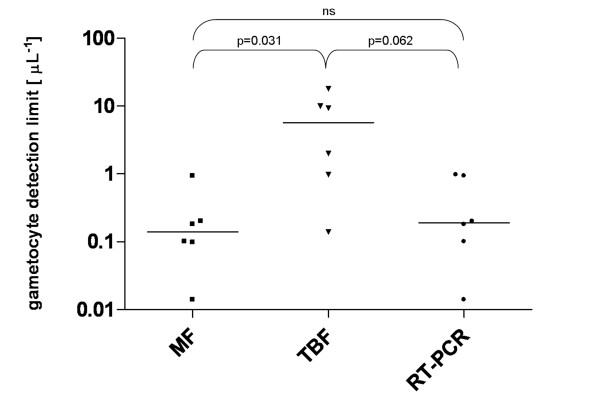
**Detection limits for gametocytes using magnetic fractionation (MF), thick blood film (TBF), and RT-PCR techniques**. Wilcoxon's matched pairs test was used as the significance test.

RT-PCR showed bands of similar sized product, to that described by Babiker *et al *[13] and Mlambo *et al *[12], and Menegon *et al *[14] in all experiments down to the range of 10^-1^–10^0 ^gametocytes μL^-1 ^(Figure 3). The limits of detection for three methods for each dilution series are shown in Figure 4.

In pairwise comparison, the limits of detection for the MF technique were significantly lower than those for the TBF technique (p = 0.031) with the same trend when comparing RT-PCR to TBF (p = 0.062). There was no significant difference between the detection limits for MF and RT-PCR.

Table 1 shows the sensitivities for each method for each of the gametocyte density ranges. Both MF and RT-PCR showed 100% sensitivity for the detection of gametocytes down to concentrations in the range 10^-1^–10^0 ^μL^-1^. At lower concentrations, the sensitivity of both techniques decreased. By contrast the sensitivity of the TBF technique was below 100% in the concentration range 10^0^–10^1 ^exhibiting 0% sensitivity in the 10^-2^-10^-1 ^range. The mean observation time was considerably less for the MF technique than for the TBF technique (see Table 1).

**Table 1:** Observed sensitivities and mean observation times (t) for the magnetic fractionation (MF), thick blood film (TBF), and RT-PCR methods for the six dilution series in the four ranges of gametocyte density.

Gametocyte density range [μL^-1^]	Mean gametocyte density (range) [μL^-1^]	MF		TBF		PCR
				
		Sensitivity [%]	t [min]	Sensitivity [%]	t [min]	Sensitivity [%]
10^1^–10^2^	57 (14–97)	100	< 1	100	2	100
10^0^–10^1^	5.7 (1.4–9.7)	100	< 1	83	17	100
10^-1^–10^0^	0.57 (0.14–0.97)	100	3	33	46	100
10^-2^-10^-1^	0.057 (0.014–0.097)	50	33	0	60	33

Detection limits of the 3 different methods are compared in Figure 4. Sensitivities and slide observation times required to detect the first gametocyte are compared in Table 1.

## Discussion

In the present study, MF showed an improved gametocyte limit of detection as compared with TBF and a similar limit to that of RT-PCR. Identification of gametocytes was easier in the thin film-like MF preparations compared with the TBF preparations. Unlike current RT-PCR assays, MF assays can easily be adapted to field use and so have potential application to clinical and epidemiological studies in which gametocyte density is a variable of interest.

Several studies have focused on determining the limit of detection for asexual parasite forms and gametocytes on TBF [15-20]. An initial scan of the TBF is often conducted to detect the presence of malaria parasites before determining parasite density. The number of fields examined in the initial scan typically varies between 30 and 500 [15-17]. The volume of blood observed in a TBF is dependent on the preparation method and the technique of the microscopist. The TBF contains less blood per field on the edges while the largest blood volume per field is found in the centre. Different studies report that scanning 100 fields on a TBF corresponds to between 0.28 and 1 μL of blood [18-20]. In the present study 100 fields on a TBF preparation corresponded to a mean of 0.16 μL blood. However, if necessary, blood volumes up to 2.8 μL were assessed.

Using Poisson statistics, it can be shown that there is an approximate 95% probability that a gametocyte will be present in an observed volume (V) of blood if the gametocyte density in the source blood is equal to 3/V. Therefore for a reliable detection of a parasite density of 1 μL^-1^, a volume of at least 3 μL has to be examined on the TBF.

Such volumes are rarely achieved in routine microscopy for malaria diagnosis. Previous studies have reported that actual detection limits are higher than the calculated theoretical limit based on Poisson statistics [19-22]. A realistic lower detection limit of 50 μL^-1 ^when examining a blood volume of 1 μL on a TBF is in accord with our findings and the literature [23].

Estimates for the limit of detection for parasites on TBF range from 10^0^–10^2 ^μL^-1 ^[20,24-27], but sensitivity is reported to drop to near zero for densities below 10 μL^-1 ^[24]. One study reported the loss of 60–80% of parasites during thick film preparation [21], but this is likely to be dependent on the particular method of slide preparation [1] and was not observed in the present study. In the specific case of *P. falciparum *gametocytes, the equivalent percentage missing or obscured on TBF has been reported to be 86% [20]. A recent study claims that TBFs underestimate parasite density by about a factor of 10 [18].

Reported limits of detection for RT-PCR vary widely from 2 μL^-1 ^[12] to 0.0052 μL^-1 ^[13]. The latter figure was derived from serial dilutions and a sample volume of 50 μL and 40% haematocrit. However, based on Poisson statistics there is only a 19% probability that a gametocyte will be present in a sample of this volume and gametocyte density.

## Conclusion

Magnetic fractionation offers advantages over other techniques of gametocyte detection with a limit of detection below that corresponding to the lowest epidemiologically relevant gametocyte densities. The MF method results in preparations enabling the detection of gametocytes at concentrations two orders of magnitude below that achievable with TBF methods. Although it takes almost an hour to conduct MF as described in the present study, the time required to identify gametocytes in the resultant preparations is much shorter than that required to examine a TBF. RT-PCR samples can be processed in batches but the method cannot compete in time efficiency with either TBF or MF techniques. Owing to the cost of the single-use columns, the price for an MF preparation is about 10 US dollars and is considerably higher than that for a TBF preparation but lower than that for an RT-PCR preparation. With magnetic deposition microscopy, the cost for a magnetically fractionated sample can be reduced to the cost of a standard thick film.

## Abbreviations

MACS: Magnetically Activated Cell Sorting; MDM: Magnetic Deposition Microscopy; MF: Magnetic fractionation; RT-PCR: Reverse Transcriptase Polymerase Chain Reaction; TBF: Thick blood film.

## Competing interests

The authors declare that they have no competing interests.

## Authors' contributions

SK did the experimental work including cell culture, magnetic fractionations, RT-PCR and slide analysis. SK, TD and TSP conceived of the study. SK, TD, TSP prepared the manuscript.
